# Neurological Manifestations of Scrub Typhus Infection in Pediatric Patient: A Case Report From Tertiary Care Hospital in Central India

**DOI:** 10.7759/cureus.53060

**Published:** 2024-01-27

**Authors:** Priyanka Singh, Vishal Shete, Abhijit Choudhary, Neeta Gade, Meena Mishra

**Affiliations:** 1 Department of Microbiology, All India Institute of Medical Sciences, Nagpur, Nagpur, IND; 2 Department of Pediatrics, All India Institute of Medical Sciences, Nagpur, Nagpur, IND

**Keywords:** rickettsial disease, scrub typhus meningitis in children, scrub typhus meningoencephalitis, scrub typhus meningitis, scrub typhus case report

## Abstract

Scrub typhus is a neglected tropical bacterial disease endemic in central India which can manifest as meningitis/meningoencephalitis in children. It is difficult to diagnose clinically, especially in the absence of eschar or rash. Scrub typhus is seldom considered the differential diagnosis of meningitis in the Indian subcontinent. Appropriate investigations can lead to early detection of infection and initiation of correct antibiotic treatment leading to better patient prognosis even when features of meningitis supervene. Here, we report a pediatric case of scrub typhus meningitis that could be saved due to timely investigations and initiation of appropriate antimicrobial agents.

## Introduction

Scrub typhus is an infectious disease caused by the bacteria Orientia tsutsugamushi. It is primarily transmitted to humans through the bite of infected larvae of Leptotrombidium mites, commonly known as chiggers. This bacterial infection is prevalent in a specific geographical region called the “tsutsugamushi triangle,” which encompasses parts of the Asia-Pacific region. India falls within this geographical area endemic to scrub typhus [1]. Within India, certain regions are particularly prone to scrub typhus cases. These include Maharashtra and Rajasthan in the west, Himachal Pradesh, Uttaranchal, and Jammu & Kashmir in the north, Meghalaya, Assam, and Nagaland in the northeast, West Bengal and Bihar in the east, and Tamil Nadu, Andhra Pradesh, Karnataka, and Kerala in the south. The abundance of dense vegetation in these areas provides an ideal habitat for chiggers, the vectors responsible for transmitting the infection, to thrive and proliferate [2]. Scrub typhus affects multiple organs. The typical manifestation of scrub typhus infection includes symptoms: fever with or without a rash, swollen lymph nodes, and the presence of an eschar (a dark, scab-like lesion) at the site of chigger bite [3,4]. In addition, other symptoms like shortness of breath, cough, headache, nausea/vomiting, and changes in mental alertness may be present. About one-third of scrub typhus patients experience complications involving multiple organs: lungs, liver, heart, nervous system, or kidneys [2].

## Case presentation

In September 2022, a five-year-old girl from the Amravati district of Maharashtra presented to the pediatric emergency department with chief complaints of fever, headache, and vomiting persisting for five to six days. On examination, the child was febrile and irritable, along with altered sensorium. Signs and symptoms suggesting raised intracranial pressure were observed. Kernig's sign and Brudzinski's sign were also positive during systemic examination, indicating the possibility of meningitis. No signs of rash, eschar, and lymphadenopathy were present. Basic blood investigations were sent, papilledema was ruled out and lumbar puncture was done. The child was started on empirical antibiotics (ceftriaxone) for suspected meningitis. Table 1 enlists the various blood and cerebrospinal fluid (CSF) investigations done for this case along with their results.

**Table 1:** List of various investigations (with their results) done for the patient Abbreviations: CRP – C-reactive protein; CSF – Cerebrospinal fluid; CBC – Complete blood count; RBC – Red blood cells; WBC – White blood cells; MCV – Mean corpuscular volume; MCH – Mean corpuscular hemoglobin; MCHC – Mean corpuscular hemoglobin concentration; KFT – Kidney function tests; BUN – Blood urea nitrogen; LFT – Liver function tests; ALP – Alkaline phosphatase; AST – Aspartate aminotransferase; ALT – Alanine aminotransferase Units: mg – milligram; L – liter; dL – deciliter; g – gram; fl – femtolitre; pg – picogram; U – units *90% cells were Lymphocytes; 10% Neutrophils; No RBCs **All mononuclear WBCs; No RBCs ***Manually verified

S. No.	Name of the Investigation/Test	Parameter (Measurement Unit)	Result	Reference range (for age group 5 – 6 years)
1.	CRP Quantitative	CRP Quantitative (mg/L)	84.5	< 5.0
2.	CSF Analysis	CSF glucose (mg/dL)	57	50 - 80
		CSF protein (mg/dL)	98	15 - 60
		CSF cell count (cells/mm^3^)	102*	0 - 5**
3.	CBC	Hemoglobin (g/dL)	10.8	11.5 - 14
		RBC (x 10^12 ^/L)	4.42	3.9 - 5.3
		WBC (x 10^9 ^/L)	7.69	5 – 17
		Neutrophils (%)	46.1	40 – 80
		Eosinophils (%)	0.4	1 – 6
		Basophils (%)	0.1	0 – 1
		Lymphocytes (%)	50.9	20 – 40
		Monocytes (%)	2.5	2 – 10
		Hematocrit (%)	33.2	34 – 40
		MCV (fl)	75.1	73 – 91
		MCH (pg)	24.5	24 – 30
		MCHC (g/dL)	32.6	32 - 34.8
		Platelet count (x 10^9 ^/L)	89***	150 – 400
4.	KFT	BUN (mg/dL)	6	15 – 39
		Serum creatinine (mg/dL)	0.3	0.6 – 1.3
5.	LFT	Alkaline phosphatase (U/L)	204	130 – 260
		AST (U/L)	381	0 – 45
		ALT (U/L)	148	0 – 45
		Albumin (g/dL)	2.7	3.5 – 5
		Total Bilirubin (mg/dL)	0.58	0.2 – 1.2
		Total Protein (g/dL)	6.5	6 – 8.3

Culture and serological investigations

Following aerobic incubation at 37°C, no growth was observed in urine, CSF, and blood cultures, indicating the absence of commonly cultivable bacterial pathogens in these samples. Peripheral blood smear examination for malarial parasites and rapid malaria card test were negative, ruling out malaria as the cause of the symptoms. However, C-reactive protein (CRP) levels were significantly elevated at 84.5 mg/L, suggesting an inflammatory response. Additionally, the Scrub Typhus IgM test was positive by immunochromatography (ICT), indicating the presence of IgM antibodies associated with scrub typhus. This finding was further confirmed by the “reactive” Scrub Typhus IgM ELISA test result for the same serum specimen. The Scrub Typhus IgM Units for this patient were 76.25; Scrub Typhus IgM Units > 11 were considered positive [5].

CSF examination

The CSF specimen was collected in a sterile container with all aseptic precautions on the same day as the date of admission. It was clear and colorless, with a volume of 0.5 mL, and no coagulum formation was observed on standing. The CSF glucose level was measured at 57 mg/dL, which falls within the normal range. However, the CSF protein level was slightly elevated at 98 mg/dL. In terms of cellular composition, the CSF cell count was determined to be 102 cells/mm^3^. Of these cells, 90% were lymphocytes and 10% were neutrophils. India ink examination was negative for capsulated organisms. Gram stain examination revealed the presence of occasional pus cells, but no organisms were observed. Subsequently, after 72 hours of aerobic incubation at 37°C, no growth was detected in CSF culture.

Blood investigations

The results of blood investigations revealed several abnormalities. Hemoglobin levels were lower than normal at 10.8 mg/dL. Complete blood count (CBC) indicated lymphocytosis, with lymphocytes accounting for 51% of the total white blood cells. Additionally, the platelet count was found to be decreased, measuring 89 x 10^9^/L. Both kidney and liver function tests were deranged, with low blood urea nitrogen (BUN) levels at 6 mg/dL and reduced serum creatinine levels at 0.3 mg/dL. Serum albumin levels were low, measuring 2.7 g/dL; alanine aminotransferase (ALT), and aspartate aminotransferase (AST) levels were elevated at 148 U/L and 381 U/L, respectively.

Treatment, outcome, and follow-up

The patient was given intravenous ceftriaxone empirically on the day of admission. However, based on the positive ICT and ELISA results for scrub typhus infection, the patient was started with doxycycline on second day. Doxycycline was given intravenously at a dose of 2.2 mg/kg twice daily. As a result, both the symptoms and biochemical markers started showing improvement within two days of doxycycline initiation. CBC, CRP, and serum electrolytes were done daily whereas kidney and liver function tests were done on every alternate day during the initial five days of starting doxycycline therapy. A repeat CSF examination was done after five days of therapy and all the parameters were found within normal limits. Intravenous doxycycline was given for eight days. Thereafter, following the positive response, the patient was discharged after nine days of hospitalization while continuing the course of doxycycline orally. At the time of discharge, the patient was advised to take half a tablet of 100 mg doxycycline twice daily for two days. Thus, the total duration of doxycycline therapy was of 10 days. A plan was implemented for regular follow-up visits at the outpatient department as and when needed, ensuring appropriate monitoring and care. The patient soon became normal on subsequent follow-up visits.

## Discussion

Scrub typhus meningitis presents as a medical emergency, necessitating immediate evaluation and treatment. Scrub typhus, characterized by an acute febrile illness, can manifest as meningitis or meningoencephalitis in approximately one-fifth of affected individuals [6]. The pathogenesis of central nervous system involvement in scrub typhus is not completely understood. It is believed to result from the direct invasion of brain parenchyma or an immune-mediated response to the infection. Neurological manifestations can range from meningitis to meningoencephalitis, and in severe cases, it can lead to coma, seizures, and focal neurological deficits. Diagnosing scrub typhus meningoencephalitis can be challenging due to its non-specific clinical features [7]. CSF analysis of patients with scrub typhus has significantly less pleocytosis, greater lymphocyte proportion, and a lesser degree of protein elevation than in cases of bacterial meningitis [6]. However, with appropriate laboratory investigations such as scrub typhus IgM ELISA, the disease can be easily diagnosed if scrub meningitis is considered as a differential diagnosis, especially in endemic regions. We diagnosed scrub typhus in this patient using Scrub Typhus IgM Microlisa by J. Mitra & Co. Pvt. Ltd, New Delhi, India, with a sensitivity of 100% and specificity of 98.6% [5]. Early initiation of appropriate antibiotics, such as doxycycline, is extremely crucial in managing scrub typhus meningoencephalitis because typhus infections in general will not respond to ceftriaxone, which is commonly used for bacterial meningitis. Prompt treatment can lead to a significant reduction in morbidity and mortality [2].

## Conclusions

Scrub typhus meningoencephalitis is a rare but important complication of scrub typhus infection. Clinically, it can be difficult to diagnose in the absence of eschar, rash, or lymphadenopathy. In endemic areas, scrub typhus should be considered as one of the differentials of aseptic meningitis. CSF analysis in scrub typhus usually shows modest elevation in the WBC count with lymphocyte predominance, a moderately elevated protein level, or a normal to low sugar level and should be differentiated from other CNS infections. Appropriate laboratory investigations can help in early diagnosis of the disease and initiation of the right antibiotics leading to better patient outcomes and decreased hospital stay.
